# Environmental determinants of male infertility: emerging threats and technological interventions

**DOI:** 10.3389/fmed.2026.1770866

**Published:** 2026-02-18

**Authors:** P. Jagadesh, T. B. Sridharan

**Affiliations:** Gene Cloning Technology Lab, School of Biosciences and Technology, Vellore Institute of Technology, Vellore, India

**Keywords:** artificial intelligence, assisted reproductive techniques (ART), environmental pollutants, male infertility, semen quality

## Abstract

Male infertility stands as a significant global concern, contributing to nearly 50% of infertility cases and affecting approximately 7% of the male population. Mounting evidence identifies environmental degradation is a major, modifiable driver. Numerous environmental contaminants, including air pollution, heavy metals, endocrine-disrupting chemicals (EDCs), microplastics, pharmaceutical contaminants, and climate change linked to deteriorating semen quality. These environmental toxins can decrease spermatogenesis and overall sperm function by triggering oxidative stress, hormonal imbalance, inflammation, and epigenetic alterations. This review highlights the increasing necessity of incorporating environmental exposure data (eco-profiles) into routine semen analysis. An integrated framework is outlined in which AI algorithms analyze multi-omics biomarkers, ranging from genomics to metabolomics, together with environmental metrics. These combined data are used to predict individual fertility risk and to guide personalized treatment strategies, particularly in the context of assisted reproductive technologies. Future studies are essential to find trustworthy biomarkers and elucidate the molecular processes that connect environmental contaminants to male infertility. As environmental toxicants intensify, the comprehensive toxicological studies are in need to enhance curative approaches and preventative strategies that ultimately aim at safeguarding male fertility.

## 1 Introduction

**Figure 1:**
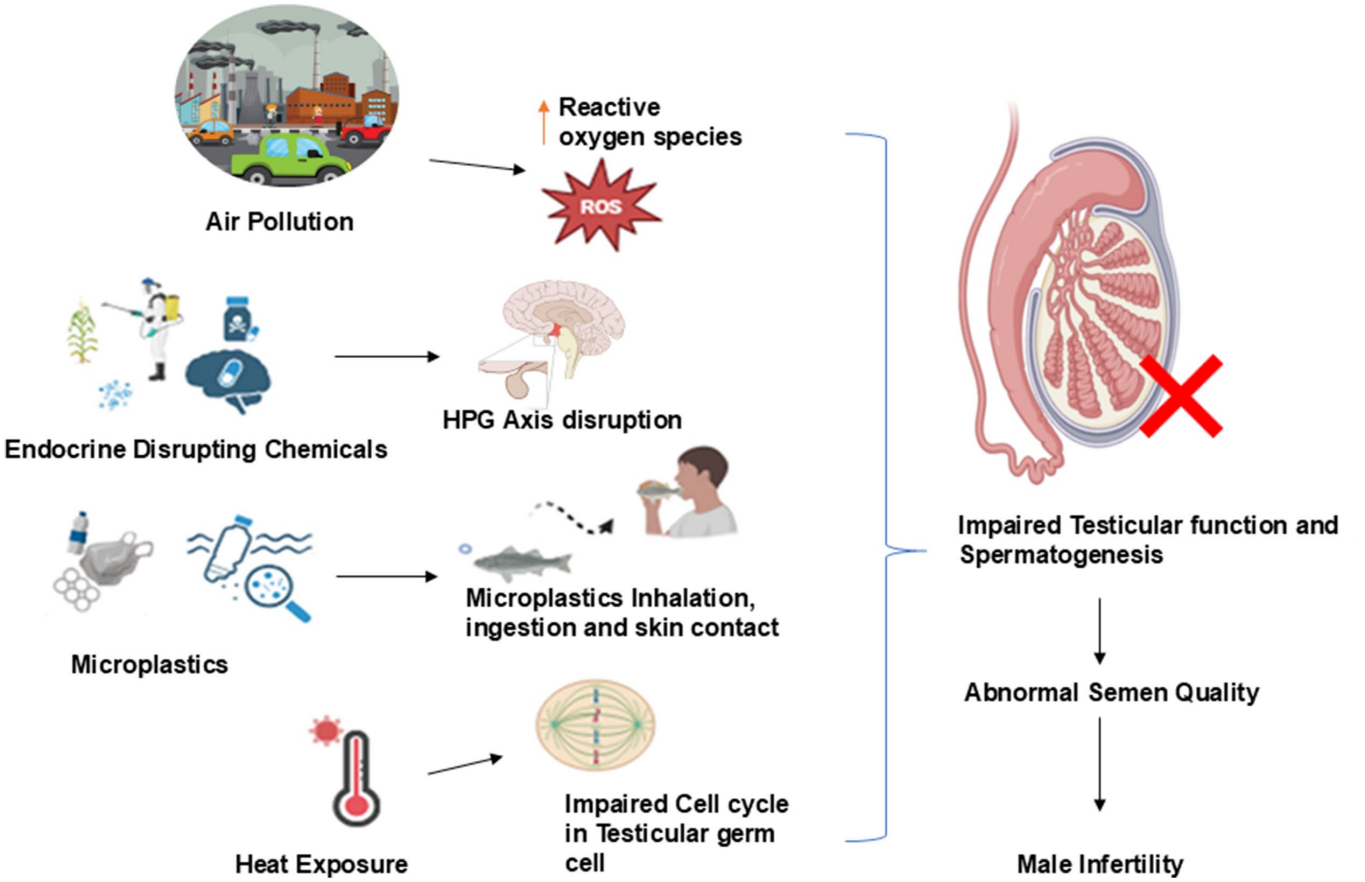
Environmental factors impacting male fertility.

Infertility is defined as the inability of a couple to achieve pregnancy even after 12 or more months of regular unprotected sexual intercourse. About 186 million individuals were living with fertility issues throughout the world. Around 1 in 6 people are living with fertility issues. Male factors contribute to around 50 % of the overall cases of infertility. It affects about 7% of men worldwide and eventually hampers millions of families (1). Male infertility is emerging as a major global health concern as the quality of semen is gradually declined over the recent decades (2–4). In our ecosystem, everything is interlinked to environmental pollution, lifestyle, and human reproductive health. From the air humans breathe, the chemicals that exposed to in the environment, lifestyle factors, and the genes inherited from parents all these pieces matter in health outcomes. Increasing pollution levels as due to heavier urbanization will alter the environment and increase the burden of male infertility. Male fertility potential is significantly influenced by environmental factors. Exposure to certain pollutants, including toxic heavy metals, pesticides, and endocrine-disrupting chemicals, has been shown to affect male reproductive health. Exposure to high temperatures and electromagnetic radiation also has detrimental effects on male reproductive function (Figure 1) (5). Exposure to environmental pollutants can be directly toxic to human semen (6). Male reproductive capacity may have declined due to other circumstances as well. However, plastic pollution is by far the most chronic menace to humanity. According to certain fundamental and retrospective scientific research, environmental pollutants, food, and obesity may all be contributing factors to this drop in sperm parameters (7). Though there is a great technological and research development in identifying biomarkers related to male infertility, semen analysis remains the primary initial diagnostic test. It continues to be the most widely used and fundamental assessment in the evaluation of male infertility. Semen analysis provides a comprehensive assessment on the male reproductive health (8). In addition to that, semen analysis offers critical insights into sperm DNA integrity, essential for fertilization, embryo development, and guiding assisted reproductive techniques (ART), including *in vitro* fertilization (IVF) or intracytoplasmic sperm injection (ICSI) (9–11). This review aims to discuss the consequential effects of environmental factors on semen quality.

## 2 Environmental factors

**Figure 2:**
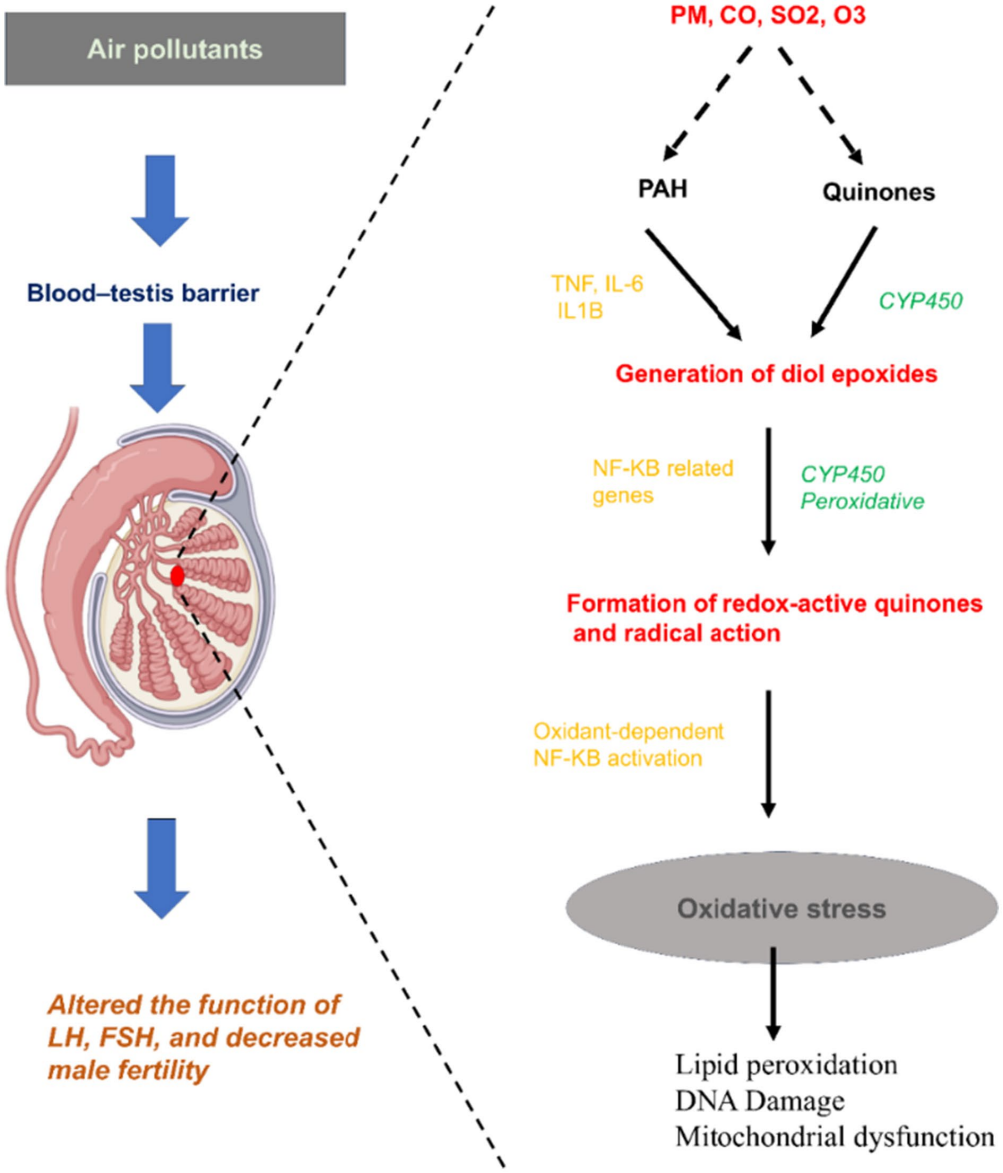
Schematic representation of air-pollution-induced oxidative stress.

Human activities have introduced several pollutants into the worldwide ecosystem. Increasing population leading to mass industrialization, deforestation, urbanization, and the exploitation of natural resources eventually leads to pollute air, land, and water. It is estimated that reproductive disorders account for about 23% of all cases of male infertility while the remaining percentage is due to environmental pollutants/toxicants (12). Compared to women, men are more exposed to environmental pollutants that are recognized as risk factors for infertility (13). Environmental pollution was observed to have an unfavorable effect on human reproductive health as it leads to deterioration of semen quality by impairing the process of spermatogenesis, steroidogenesis, sperm function, and Sertoli cell damage, thereby leading to decreased male fertility (14, 15). Exposure to environmental pollutants, such as heavy metals, pesticides, endocrine-disrupting chemicals (EDCs), and air pollution, has been strongly associated with impaired sperm parameters and hormonal imbalances (16). The increase in the environmental quality deterioration can be a great hazard to male fertility potential (17). For instance, ambient airborne pollutants particulate matter (PM), carbon monoxide, sulfur dioxide, and ozone can penetrate the pulmonary systemic circulation and access the blood-testis barrier. The molecular toxicity of air pollution, particularly particulate matter and polycyclic aromatic hydrocarbons (PAHs), extends beyond simple physical irritation. Upon inhalation, PAHs are bioactivated by cytochrome P450 (CYP450) enzymes, converting them into highly reactive electrophiles such as diol-epoxides and redox-active quinones. These metabolites act as potent molecular triggers for the NF-κB signaling pathway. Specifically, the accumulation of these electrophilic species up-regulates NF-κB-responsive genes, driving the surge of pro-inflammatory cytokines including TNF-*α*, IL-6, and IL-1β. This specific inflammatory cascade does not merely cause oxidative damage (Figure 2), it actively disrupts the blood-testis barrier and Leydig cell function, directly impairing the hormonal signaling of luteinizing hormone (LH) and follicle-stimulating hormone (FSH) required for spermatogenesis (18–20).

Although environmental determinants of male infertility originate from diverse sources including airborne particulates, heavy metals, endocrine-disrupting chemicals, microplastics, and temperature changes they cannot be an isolated threat (21). These factors operate within a unified toxicological framework. Despite entering the body through distinct pathways such as inhalation, ingestion, or dermal contact, these pollutants converge on shared cellular processes that impair spermatogenesis and sperm function. Oxidative stress characterized by an imbalance between the generation of reactive oxygen species (ROS) and antioxidant defences. Air pollutants (PM2.5, NO₂), heavy metals (lead, cadmium), and microplastics have each been associated with excessive ROS production, that leads to lipid peroxidation, mitochondrial dysfunction, and sperm DNA fragmentation (22). Beyond oxidative injury, these exposures act synergistically on endocrine regulation of male fertility. The hypothalamic pituitary gonadal (HPG) axis is affected not only by endocrine-disrupting chemicals such as phthalates, but also by heavy metals and thermal stress, leading to reduced testosterone production and altered gonadotropin signalling. These insults are further accompanied by epigenetic alterations, including DNA methylation changes, that influence genes essential for spermatogenesis (23). These converging mechanisms create a “cocktail effect,” producing cumulative reproductive toxicity that exceeds the effect of any single pollutant.

### 2.1 Air pollution

Air pollution is considered the most serious environmental concern on Earth, as 99% of the world population were living in the places where WHO air quality guidelines levels were not met (24). The development in urbanization, population, and industrialization has significantly increased ambient air pollution in urban cities, both locally and globally, causing some of the most serious air quality challenges. At once, India was considered as the world’s second most polluted country (25). The elevated levels of air pollution in many regions of the world were profound enough to have genuine impacts on human health and have also led to numerous diseases, including respiratory (26), cardiovascular (27), cancer (28), liver (29), and reproductive diseases (30, 31). The sources of air pollution are classified into two categories: natural and anthropogenic sources, which include industrial emissions, motor vehicle exhaust, dust from construction and mining, residential activities like burning of coal, and natural sources, such as wood, wildfire, volcanic eruptions, etc. (32). Pollutants that are emitted directly into the atmosphere are termed primary pollutants, including carbon monoxide (CO), Sulphur dioxide (SO_₂_), nitrogen dioxide (NO_₂_)_,_ and particulate matter (PM). Secondary pollutants are formed through interactions with other components in the atmosphere. Some of the secondary pollutants include ozone (O_₃_) and secondary particulate matter (sulphates, nitrates, ammonium salts, and secondary organic aerosols). Also, many pollutants coexist and vary spatially and temporally, hence it is challenging to separate their effects (33). Numerous recent research studies have shown the adverse effects of air pollution on human reproductive health. Air pollutants, when inhaled, have their absorption and distribution depend on the mode of entry and solubility. It was determined that increased ROS can impact the semen quality parameters through different pollutants as showed in Table 1. The most explored mechanisms are inflammation, the induction of apoptosis, destruction of the blood-testis barrier, and oxidative stress (34, 35). Oxidative stress can be defined as the state of imbalance in the ROS production and antioxidant activity (36). It is well established that most of the air pollutants have different mechanisms but ultimately end in the production of ROS. Excessive ROS production amplifies the recruitment of proinflammatory cytokines, thereby inducing inflammation, oxidative stress, and lipid peroxidation; increases the binding of PAHs to their receptors; or interacts with PAHs to trigger DNA strand breaks and apoptosis. All these pathways contribute to the pathophysiology of diminished male reproductive potential (37, 38).

**Table 1:** Summary of epidemiological studies investigating the impact of air pollutants on semen quality.

References	Location	Period	Study design (sample size)	Pollutants	Mechanism	Key findings
(158)	China	2019–2022	Case–control (27,014)	NO_2_, PM10, PM 2.5, and SO_2_	Oxidative stress and inflammation	Increased exposure to air pollutants leads to abnormal semen volume and morphology
(186)	China	2014–2020	Large scale and multi-center study (78,952)	SO_2_, NO_2_, O_3,_ and CO.	Oxidative stress	Exposure to gaseous air pollutants leads to decreased sperm concentration Progressive motility and total motility.
(187)	China	2019–2023	Retrospective study (2,276)	PM10, O_3_, and CO	Oxidative stress	Exposures associated with reduced semen volume, progressive and total progressive motility
(188)	China	2019–2021	cross-sectional (1,515)	SO_2_, NO_2,_ and O_3_	Cytotoxicity	SO₂ showed the strongest negative impact on semen quality, indicating its key role in pollution-related reproductive harm.
(189)	Vellore, India	2023–2024	Clinical Prospective Research study (94)	PM2.5 and PM10	Oxidative stress	Ambient particulate matter exposure linked to reductions in sperm concentration, motility, and morphology across multiple studies.
(190)	Salt Lake Valley, United states	2002–2016	Cohort study (74)	PM2.5 and PM10	Epigenetic modifications	Seasonal air pollution exposure induces hypomethylation at specific regions of the sperm epigenome.
(191)	China	2018–2019	Retrospective, cohort study (1,168)	CO, SO_2_, NO_2_, O_3_, PM_10_, and PM_2.5_	Developmental toxicity	Ambient air pollutants exposures during sperm development may have an adverse effect on semen quality, particularly for sperm count and motility
(192)	China	2014–2022	Retrospective longitudinal study (5,114)	PM_2.5_, PM_10_, NO_2_, SO_2_, O_3_, and CO	Oxidative stress	Air pollutants have negative impact on sperm motility and semen volume, and these impacts were less pronounced in spring and autumn
(193)	China	2013–2018	Retrospective study (686)	PM2.5, PM10, and CO	Impaired spermatogenesis	Chronic exposure to PM10 and PM 2.5 was linked to decreased sperm concentration and reduced motility which indicates impaired spermatogenesis
(194)	Utah, Unites States.	2005–2017	Retrospective cohort study (21,563)	Industrial air pollutants	Endocrine disruption	Chronic low-level exposure was linked to poorer semen quality increases odds of azoospermia and decreased total motility and semen volume

Air pollutants, especially the particles that are >2.5 and <10 μm in diameter, which are commonly known as PM_2.5_ and PM_10,_ can disrupt the blood-testis barrier and cause oxidative stress. Recent studies have shown that exposure to PM_2.5_ and PM_10_ has significantly decreased the sperm concentration, motility during the entire spermatogenesis period (39). The major air pollutant, O_₃_ exposure, is associated with declined sperm concentration (40). The PM and CO are associated with declined sperm count, motility, and elevated morphological defects (41). Exposure to CO and SO_₂_ appeared to influence sperm motility parameters and influence semen quality throughout spermatogenesis (42). A recent study reveals that exposure to ambient SO_₂_ was negatively associated with all semen quality parameters except for the sperm concentration progressive motility (43). A recent Meta-analysis reveals that the higher exposure to outdoor air pollution is associated with abnormal semen quality and increased DNA fragmentation index (44). A notable trend observed that predominance of studies linking air pollution and semen quality originate from China, shows high industrial activity, and availability of large clinical cohorts. However, the predominance of data from a single region raises questions about global generalizability. Air pollution composition varies geographically; for example, Pollution profiles differ significantly by geography; for instance, particulate matter in rapidly industrializing regions often contains higher concentrations of heavy metals (arsenic, lead) and sulfates from coal combustion, whereas pollution in Western urban centers is primarily driven by nitrate-rich vehicular emissions (45). These differences influence particulate matter constituents, including metals, PAHs, and secondary organic aerosols, which may have biological effects on the male reproductive system. In addition, inter-population variation in genetic background, particularly in oxidative stress–response genes and xenobiotic-metabolizing enzymes, may modify individual susceptibility to pollution-related reproductive toxicity. This geographical bias limiting the global generalizability of the findings, so this highlights the need for similar studies in other regions, especially in countries with rising pollution levels and limited reproductive health data, such as in South Asia, the Middle East, and Africa. Diverse environmental exposures, genetic backgrounds, and lifestyle factors across populations could lead to different susceptibility patterns and outcomes, which are currently underrepresented in the literature.

### 2.2 Heavy metals and male reproductive dysfunction

Several metals and metalloids (elements that lie in the intermediate state between metals and non-metals) are essential for living organisms, playing crucial roles in cell division and metabolism while also facilitating endocrine signaling between organs. However, excessive concentrations of certain metalloids can lead to heavy metal toxicity (46). Heavy metals are naturally occurring elements with a large atomic weight and density of at least 5 times greater than that of water (47). Heavy metals such as cadmium (Cd), lead (Pb), chromium (Cr), and mercury (Hg) are among the most dangerous for male reproductive health and fertility. Chronic exposure to these metals can potentially impact male fertility by causing severe oxidative stress and cellular toxicity, even at low concentrations (48–50).

#### 2.2.1 Cadmium (cd)

Cadmium is a highly toxic heavy metal that occurs naturally in the Earth’s crust but is also introduced into the environment through industrial processes such as mining, electroplating, and the use of cadmium-containing fertilizers (51). Cadmium is widely distributed in soil, water, and air, posing significant environmental and health risks. Cadmium in the environment enters the human body primarily through inhalation, ingestion, and dermal exposure, with the most concerning exposure route being through contaminated food, particularly crops grown in cadmium-rich soils (52). Cadmium is a heavy and toxic trace metal without an essential role in the human body. The half-life of Cd in the blood is 3-4 months (53). This heavy metal will accumulate in the testicular tissues. The accumulation of Cadmium on the testicular tissues has a detrimental impact on spermatogenesis (54). Cadmium exerts multiple detrimental effects on reproductive health through various mechanisms, including disruption of the HPG axis, structural damage to the blood-testis barrier and testicular vasculature. Additionally, cadmium induces oxidative stress in both somatic and germ cells, triggers inflammation, promotes apoptosis in germ cells, and exerts epigenetic modifications (55, 56). At the cellular level, cadmium exposure has been shown to suppress the Akt signaling pathway (protein kinase B), a critical regulator of cell survival, while simultaneously activating p53. This shift in downregulating survival signals while upregulating apoptotic factors accelerates germ cell death and structural abnormalities in sperm. Furthermore, cadmium exerts long-term toxicity through epigenetic silencing, even recent studies indicate that it induces hypermethylation of the metallothionein 1A (MT1A) gene promoter (57). This specific epigenetic modification reduces the testis’s ability to produce metallothionein, proteins crucial for detoxifying heavy metals, thereby locking the reproductive system in a cycle of increased susceptibility to oxidative stress. Beyond its reproductive toxicity, it significantly impacts human health by disturbing intracellular calcium homeostasis, generating oxidative stress, and interfering with critical cell signaling pathways, ultimately leading to cellular dysfunction (49). Cadmium exposure has been associated with impaired spermatogenesis, DNA fragmentation, and reduced sperm motility, with studies showing a direct correlation between elevated cadmium levels in the blood or semen and poorer semen quality (58). Furthermore, a recent study conducted has revealed that cadmium levels in seminal plasma were higher in infertile men with lower sperm quality, higher oxidative stress, and higher MT1A methylation (59). A recent meta-analysis supports these findings that infertile men had higher seminal cadmium levels than fertile men (60).

#### 2.2.2 Lead (Pb)

Lead is the second most toxic metal, which comprises 0.002% of Earth’s crust. It is naturally found in a very limited amount, but it is mostly produced due to human-made industries, automobiles, batteries, etc., due to which the same human and its environment are getting affected by the lead pollution. Humans ingest 30-60% of lead primarily, and it also gets absorbed by the body (61). It is released at high extents in the soil, water, and air through industrial waste and from household things such as batteries, poisoning may occur on frequent exposure and poses a significant threat to human health, including male reproductive health. Lead exposure occurs primarily through ingestion of contaminated food and water, inhalation of polluted air, and direct dermal contact with contaminated surfaces (62). Once if it gets through the human body, lead accumulates in soft tissues and bones, disrupting cellular functions and inducing oxidative stress, a key mechanism in male infertility (63). Recent studies have demonstrated that even low-level lead exposure can impair sperm parameters, including motility and morphology, and disrupt hormonal balance, particularly testosterone production by targeting the HPG axis (15). Moreover, its ability to induce DNA damage in sperm underscores its potential to affect fertilization and embryonic development (60). Lead accumulates in the testicular tissue and disrupts the HPG axis, reducing testosterone synthesis and spermatogenesis (64). It also induces oxidative stress, damaging Sertoli and Leydig cells critical for sperm production (65). Elevated blood lead levels correlate with decreased sperm concentration, motility, and morphology abnormalities (58). In addition to that, a meta-analysis report reveals that lead exposure is associated with lower semen volume, decreased sperm count and vitality (66). Furthermore, supporting the fact that lead exposure seriously affects the sperm functions in males, resulting in poor-quality semen and hence, male infertility.

#### 2.2.3 Mercury (hg)

Mercury is naturally present in the Earth’s crust; however, anthropogenic activities, including mining and the combustion of fossil fuels, have resulted in extensive global mercury contamination. Mercury released into the atmosphere ultimately deposits into aquatic environments or terrestrial surfaces, where it may be transported into water bodies (67). Upon deposition, specific microbes can convert it into methylmercury, a very poisonous variant that accumulates in fish, shellfish, and animals that consume fish (68). Notably, the methylation and subsequent bioaccumulation of mercury in the aquatic food chain as methyl mercury elevates the entry into the food web (69). United States Environmental Protection Agency (USEPA) estimates that the annual global mercury emissions from anthropogenic sources are approximately 2,220 metric tons per year (214). Mercury is eventually released into the environment primarily through industrial processes, mining, and coal combustion. It leads to increased mercury levels in aquatic ecosystems (70, 71). Human exposure to mercury primarily occurs through occupational settings, dental amalgams, and consumption of contaminated fish and seafood (72). The bioaccumulation of mercury in aquatic organisms especially fish, is a major route of exposure for humans (73). Its toxic effects extend to male reproductive health, where it adversely effects on testicular function, impairs spermatogenesis, and induces oxidative stress through mechanisms such as elevating the Reactive oxygen species concentration, mitochondrial dysfunction, and cellular apoptosis which severely affects the reproductive health (74, 75). Additionally, compared to fertile people, infertile subjects with unexplained infertility had greater amounts of mercury in their hair, blood, and urine. It was found that the blood mercury levels are higher in infertile males compared to fertile males. Higher blood mercury levels were significantly associated with sperm morphology defects (76). Exposure to mercury caused abnormal sperm motility and morphology as well as damage to the sperm DNA, leading to the reduction in sperm quality (77). In addition to that, mercury levels are also linked to higher rates of poor reproductive outcomes as well as a higher incidence of hormonal abnormalities (71). Furthermore, its exposure could damage Leydig cells, seminiferous tubules, and testicular degeneration (66). Thus, mercury and other heavy metal exposure has the potential to affect male reproductive functions, which adversely affects the semen quality as showed in Table 2. However human studies correlating the mercury exposure levels and sperm quality is explored less. More research is required to understand deeply the mechanism behind the impact of mercury on reproductive health and to develop the effective management strategies. Hence, continued monitoring and research efforts are essential to ensure public health and establishing policies for enhancing environmental quality.

**Table 2:** Heavy metal exposure with semen quality outcomes across various geographic locations.

References	Study groups	Country	Biological matrix	Heavy metal	Mechanism	Key findings
(195)	Male tea garden workers (*n* = 200) and samples from age-matched donors as control group (*n* = 200)	Southern Assam, India	Semen	Pb, Cd	Apoptosis (p53 activation)	High heavy metal levels activate p53 and suppress Akt pathway, leading to sperm abnormalities and increased seminal oxidative stress.
(196)	Male partners in couples from a reproductive medicine center (*n* = 1,020)	Wuhan, China	Urine	As, Cd, and Pb	Oxidative stress (lipid peroxidation)	Higher urinary As, Cd, and Pb associated with increased 8-isoPGF2α and 8-OHdG, mediating reduced motility and normal morphology in sperm
(197)	Non-smoking males visiting infertility clinics (*n* = 333)	Wuhan, China	Urine	Cd	Synergistic toxicity	Cd modified the association between polycyclic aromatic hydrocarbons (PAH) and pyrene with seminal quality parameters.
(198)	Samples were collected from clinically diagnosed 130 oligozoospermic male (*n* = 120)	Pakistan	Blood and Semen	Cd, Cr and Pb	Endocrine disruption	Heavy metals from Indus River fish contribute to oligozoospermia and infertility
(199)	Infertile patients (*n* = 101)	Iraq	Serum and semen	Cd and Pb	Inconclusive	The results showed a weak link between lead and cadmium levels in the blood and sperm count.
(200)	Male partners of couples investigated for infertility (*n* = 300)	Sri Lanka	Semen	Pb	DNA fragmentation	Pb-positive men showed higher sperm DNA fragmentation with a significant positive correlation to seminal Pb levels
(201)	Men living in rural or industrial areas. (*n* = 400)	South Italy	Semen and blood	Cd and Pb	Motility impairment	Cd and Pb in semen were suggestively linked to reduced sperm count and motility, with weak detrimental effects on overall sperm quality.
(202)	Men attending the Prenatal Diagnosis Laboratory (*n* = 100)	Iran	Semen	Cd and Pb	Oxidative stress	Higher seminal Pb and Cd levels were linked to poor sperm parameters, elevated MDA, and reduced antioxidant enzyme activity.
(203)	Reproductive-aged men (*n* = 413)	United States	Semen and urine	Cd and Cu	Genotoxicity (DNA damage)	Cd and Cu showed detrimental effects on semen quality, increasing DNA damage or reducing count.
(204)	Infertile men (*n* = 130)	Nigeria	Semen	Cd, Fe and Pb	Oxidative Stress	Higher seminal Cd, Fe, and Pb levels were linked to reduced antioxidants, lower sperm count, and poor motility.

## 3 Endocrine-disrupting chemicals (EDCS)

Endocrine-disrupting chemicals (EDCs) are exogenous substances that could mimic endogenous hormones that are responsible for self-balance, reproduction, development and behavior of natural hormone synthesis, secretion, and transport. Hence, it can interfere with the endocrine system, leading to adverse health outcomes and effects on male reproductive axis causing infertility (78). Common EDCs such as phthalates, bisphenol A (BPA) and pesticides are prevalent in various consumer products, leading to widespread human exposure. Recent studies have demonstrated that higher urinary levels of these chemicals correlate with impaired semen parameters and increased oxidative stress, suggesting a detrimental effect on male fertility (79, 80).

### 3.1 Phthalates

Phthalates are synthetic organic chemicals that are used in almost wide variety of products in industries as solvents, plasticizers, and additives in polyvinyl chloride (PVC) plastics or personal care products (PCPs) (81). The difference in molecular weight of phthalates makes it to be useful for different purposes. The high molecular weight phthalates (for example, di-isodecyl phthalate, benzyl butyl phthalate (BBP), and di-2-ethylhexyl phthalate) are used primarily in PVC polymers and plastisol applications, plastics, food packaging, and food processing materials, vinyl toys and vinyl floor coverings, and building products. The low molecular weight phthalates (for example, diethyl phthalate, di-isobutyl phthalate, and di-n-butyl phthalate) are often used in non-PVC applications, such as certain dietary supplements, personal care products and enteric-coated tablets (82). Though the phthalates are essential in useful commercial products, both types are toxic. High molecular weight phthalates are considered more harmful due to their stronger anti-androgenic effects, leading to reduced sperm quality and testicular damage. However, low molecular weight phthalates also cause significant harm, particularly in disrupting hormone function (80, 83). Phthalates have been implicated in compromising semen quality through several mechanisms. One primary pathway involves endocrine disruption, where phthalates interfere with hormonal balance, particularly by reducing testosterone levels. This hormonal imbalance adversely affects spermatogenesis, leading to decreased sperm count and motility. Emphasizing the negative effect on male reproductive health, a systematic review and meta-analysis found that exposure to phthalates and their metabolites is linked to a decrease in sperm quality (84). Furthermore, demonstrated to cause oxidative stress in the testes are phthalates. Further compromising sperm function is this oxidative environment’s destruction of sperm DNA and cellular membranes. Studies show that phthalate exposure lowers sperm motility, capacitance, and the acrosome reaction, therefore lowering fertilization rates and so compromising embryonic development (17). Many human research has been done on connections involving phthalate exposure and semen quality and/or circulating hormone levels among adult men. Among studies of semen quality parameters, a systematic review of the human epidemiological data concluded that there was moderate to robust evidence for reductions in semen quality in relation to phthalate exposure levels on the basis of consistent findings across up to 14 different studies (83). Delayed time to pregnancy in relation to male phthalate exposure or sperm DNA damage and sperm aneuploidy is seen for various reproductive endpoints in adult men (79). Furthermore, investigations have shown that phthalates can change the expression of genes important for reproductive purpose, therefore compromising the structural integrity and functionality of sperm. These disturbances taken together draw attention to the possible risk phthalate exposure to male fertility presents (85–87).

### 3.2 Bisphenol A

Bisphenol A (BPA) is a chemical molecule, widely utilized in many daily use products including containers to line food and beverage, thermal sheets, dental sealants, fillers and cooking utensils. BPA is also present on the internal coating of cans used in canned food which is considered as the main route of exposure. Reutilization and exposure of those containers to temperatures greater than 70 °C results in BPA leakage to food and beverage which ultimately leads to the dietary ingestion of BPA to humans (88). However, the risk of exposure through inhalation and skin contact, especially through thermal paper is also considerable. BPA induces DNA damage by elevating intracellular ROS levels, resulting in DNA breakage and enhanced migration of DNA towards the tails from the sperm nucleus (80). By mimicking the estrogen, BPA throws off hormonal equilibrium. It messes with the androgen receptor system, which lowers testosterone and generates less sperm (89). Recent research has underlined the negative consequences of EDCs especially BPA on sperm quality and general reproductive health as showed in Table 3, implying that these substances help to explain the rising male infertility frequency.

**Table 3:** An overview of recent research linking endocrine disrupting chemicals exposure to altered semen quality.

EDCs	Population	Country	Sample type	Outcomes	References
Bisphenol A and phthalate metabolites	671 men at infertility clinic	Italy	Urine and semen	Semen volume was positively associated with BPA levels and Sperm concentration had a significant inverse relationship with phthalates levels.	(205)
Phthalates	403 male participants	China	Semen	There is dose-dependently decrease in nine motility/kinematic measures; mediation analysis showed ~10 % of effect via androstenedione suppression	(206)
Bisphenol A	556 young adults aged 18–20 years	Denmark	Urine and semen	No associations between urinary bisphenol concentrations and semen quality were found.	(207)
Chlorpyrifos and other priority pesticides (in-vitro screen)	Human donor- sperm	South Africa	Semen	All concentrations of chlorpyrifos produced dose-dependent losses in motility/kinematics and increased Reactive oxygen species, indicating direct toxic action on sperm function	(208)
Bisphenol A and BPS.	984 men attending infertility clinic	China	Urine and semen	Higher exposure to individual BPA, BPS, and bisphenol mixtures was associated with impaired semen quality.	(209)
Glyphosate	128 infertile men	France	Blood and semen samples	Glyphosate detected in 62 % of samples; levels four-fold higher in seminal vs. blood plasma and positively correlated with oxidative-DNA damage	(210)
Mixed agricultural herbicides and fungicides	299 men who were not exposed to pesticides.	China	Blood and semen	Mixture-wide analysis identified three pesticides as top contributors to reduced total count and progressive motility.	(211)
BPA’s and Phthalates	98 men	United Kingdom	Urine and semen	Mixture explained 44 % of variance in reduced sperm concentration; phthalate fraction a major driver of the effect size	(212)
Bisphenol A (BPA)	116 patients	Poland	Semen	BPA exhibits a negative correlation with sperm concentration, total sperm count, and normal semen morphology. No connections were detected between BPA levels and total or progressive motility, as well as steroid hormones.	(213)

### 3.3 Pesticides

Pesticides are compounds used to either prevent, eradicate, or manage insects, weeds, fungus, and rodents. They are extensively applied in residences, public health, and agriculture to preserve crops, lower disease spread, and uphold hygienic conditions (90). Excessive or improper use can lead to release of pesticide compounds to the environment such as organochlorines, organophosphates and pyrethroids, etc. Recent scientific studies have established a significant link between pesticide exposure and male infertility, particularly concerning semen quality. Pesticides significantly impact male reproductive health primarily via endocrine disruption and oxidative stress. Numerous pesticides function as endocrine-altering chemicals (EDCs), disrupting the body’s hormonal systems. Organochlorine pesticides and their metabolites can bind to androgen receptors, so suppressing testosterone activity and altering the hormonal regulation of spermatogenesis (91). A thorough meta-analysis has shown that exposure to organophosphate pesticides significantly diminishes sperm count, concentration, motility, and normal morphology (92). The pesticides have direct harm to spermatozoa, as it interferes with hormonal regulatory processes leading to impairment of Sertoli and Leydig cell activities. Additionally, exposure to various pesticides, including organochlorines and organophosphates, is associated with a tangible decline in semen quality (93).

## 4 Dietary microplastics and reproductive health

**Figure 3:**
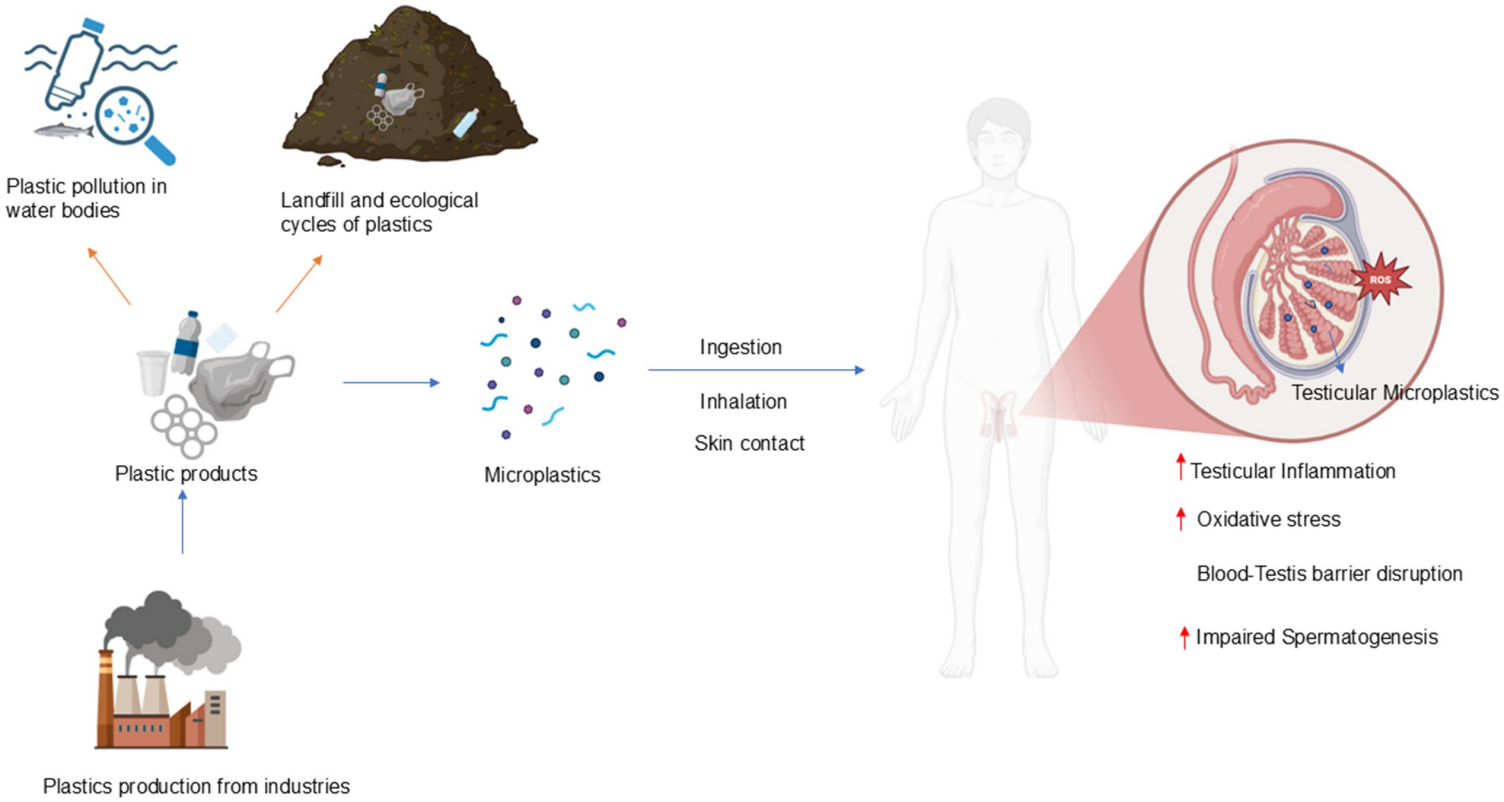
Effect of MP-induced human male reproductive toxicity.

Over the recent years, microplastics have become a major environmental and health concern worldwide (94). Plastics which are having size smaller than 5 mm is considered as microplastics (MPs). They are widely distributed in the environment, raising concerns about their long-term human health impacts. Recent evidence associates MPs with inflammatory reactions, oxidative stress, and cellular malfunction, which may lead to reproductive toxicity, gastrointestinal problems, neurotoxicity, and cardiovascular hazards (95). MPs are tiny enough to enter an organism fast, disrupt regular physiological processes (96). Since humans are at the top of the food chain likely to experience higher cumulative exposure and associated health impacts from microplastic contamination (97). An estimated 5 to 13 million tons of plastic debris are thought to reach the seas annually, where it is then consumed by aquatic life, including fish and crustaceans, and eventually makes its way up the food chain into the human diet (95, 98). Their diminutive size and ability to absorb various toxic substances, including heavy metals, can result in intestinal damage upon ingestion (99). The prevailing opinion is that they pose no threat to human health or, at most, a negligible risk. Nonetheless, direct interaction with MPs may provide adverse health effects and infertility could be one of them (Figure 3) (100). Recent studies indicate that MPs may serve as an efficient vector for the dietary absorption of polymer additives, hence exacerbating their detrimental effects on human health (101). Comprehending the dynamics of MPs bioaccumulation and trophic transfer is essential for evaluating ecological and human health hazards and developing mitigation methods.

### 4.1 Effects of microplastics on semen quality

Due to their diminutive size, microplastics can rapidly infiltrate an organism through the food chain, negatively impacting normal physiological activities. The quality of human semen has been eventually decreased from the last few decades as a result of urbanization (102). The mechanism by which it enhances the reproductive toxicity is by creating oxidative stress (103). The toxicity of microplastics is contingent upon size, with the potential for ROS formation escalating with larger plastic particle dimensions (104). Initially, it elevates the concentration of reactive oxygen species in bodily tissues and cells. Furthermore, it modifies the antioxidant systems, including Superoxide dismutase (SOD), Catalase (CAT), and glutathione (GSH), resulting in increased ROS levels that adversely affect spermatogenesis and reduce fertility (103). Recent research has identified the presence of MPs in both testes and semen, with an average concentration of 0.23 ± 0.45 particles/mL in semen and 11.60 ± 15.52 particles/g in testes (105). A recent study identified MPs ranging in size from 2 to 6 μm, suggesting their potential to breach the blood-testis barrier and induce inflammation (106). This may facilitate the uptake and release of MPs via endo- and exocytosis by macrophages within the testes, thereby disrupting spermatogenesis (107). In a study assessing the reproductive toxicity of microplastics, drinking water containing 5 μm polystyrene microplastics was administered to young male mice (1-5 weeks) during a 35-day period. After exposure the ratio of viable spermatozoa to the total number of sperm in the epididymis was found to have significantly decreased (108). Histological assessment of the testes revealed detachment of germ cells, a decrease in spermatid counts, and structural damage to the germinal epithelium (109). A subsequent investigation corroborated similar findings. Mice were subjected to oral gavage of polystyrene microplastics with diameters of 0.5, 4, and 10 μm for 28 days, resulting in bioaccumulation within testicular tissue, inflammatory responses, alterations in sperm morphology, impairment of testosterone biosynthesis, disruption of spermatogenesis, and weight reduction (110). Notably, *in vitro* studies revealed that polystyrene MPs could enter Leydig, Sertoli, and germ cells, indicating that these reproductive abnormalities could be caused by direct cellular contacts. Data still indicates that ingested MPs may accumulate in the testes and other mammalian tissues, however it is still in the early stages (111). In animal studies, this build-up has been associated with decreased semen quality, most likely due to inflammation and oxidative stress-induced damage. Several studies have demonstrated that it can translocate from the gastrointestinal tract to tissues, potentially affecting metabolism, immunity, and reproduction in aquatic organisms (112). Furthermore, these plastic particles act as carriers for hydrophobic pollutants and microbial infections, intensifying their toxicological impact (113). To present, only a limited number of research have been undertaken on the possible effects of MPs on the human reproductive system (108–111, 114, 115). Further empirical evidence is required on the entry of MPs into the human body, the concentration in human tissues (such as testes, epididymis and blood), the correlation of this concentration with semen quality, the threshold that induces harm to the male reproductive system, and the precise mechanisms via which reproductive system damage occurs (115).

## 5 Role of climate change in declining sperm quality

Climate change is significantly impacting global ecosystems, with increasing ambient temperatures posing a serious threat to human health. Investigations on the impact of climatic circumstances on semen quality are gaining significance, especially when environmental alterations are linked to human health issues, including infertility. Semen quality has been influenced by various climate-related factors, including thermal stress, atmospheric pollution, radiative contamination, intense precipitation, flooding, and aridity (38). These factors can subsequently affect male fertility. Animal study demonstrates that increased temperatures can adversely affect sperm parameters, particularly by reducing sperm concentration and motility (116). Climate change exacerbates these difficulties by exerting further strain on global agricultural systems, ultimately undermining access to healthy and nutritious meals (117). The EAT-Lancet Commission’s advocacy for plant-based diets to address climate change could provide advantageous outcomes for both the environment and human health (118). However, it is essential to ensure that these diets are adequately nourishing to prevent any potential detriment to semen quality and reproductive health (119). Fertility is not only a binary characteristic that is immutable, in contrast to viability thresholds (120). Quantitative fertility decline at intermediate temperatures seems to precede complete sterility at elevated temperatures (91). The influence of ambient heat stress on male reproductive function is a crucial although often overlooked consequence. Increased temperatures can interfere with testicular thermoregulation, hinder spermatogenesis, and lead to hormonal imbalances, hence diminishing reproductive potential (121).

### 5.1 Global warming and male fertility

Rising average local temperatures and the increasing frequency of heatwaves exemplify the swiftly evolving impacts of climate change on the global thermal environment (122). These changes are continuing to have serious effects on the distribution and abundance of natural populations and species, and they possess a serious threat to biodiversity (91). Climate change has emerged as a significant factor influencing male reproductive health (123). The core temperature is regulated above all other physiological functions in mammals, which are homeotherms with body temperatures between 35.8 °C and 39.8 °C (124). The specific thermoregulatory system of the suspended scrotum in men sustains an intratesticular temperature slightly below body temperature, essential for spermatogenesis. Superoxide dismutase, catalase, and glutathione peroxidase are antioxidant defenses activated by heat-induced oxidative stress in the testes, mostly via lipid peroxidation and ROS (125). It has been observed that high scrotal temperatures might result genetic and morphological damage, such as DNA damage, decreased sperm viability, and altered steroidogenesis (126). Elevated ambient temperatures, particularly during heatwaves, have been linked to decreased semen quality. A retrospective study in Argentina (2005–2023) involving over 54,000 men found that exposure to prolonged heatwaves during spermatogenesis led to significant reductions in sperm concentration, motility, and normal morphology (127). Statistical research has been conducted between 2000 and 2019 to investigate the global relationship between temperature change and the age-standardized prevalence rate (ASPR) of male infertility. The researchers found a U-shaped association using geographic detectors and restricted cubic spline curves, showing that higher male infertility ASPR is linked to both high and low ambient temperatures with the lowest prevalence noted at 16 °C (128). In particular, geographic locations, such as Southeast Asia and the Middle East, and those with higher Socio-demographic Indexes (SDI) showed a stronger correlation. According to Shared Socioeconomic Pathways (SSP) models, future projections indicate that rising temperatures might contribute to even higher frequency of male infertility (129). Elevated scrotal temperatures disrupt spermatogenesis by inducing oxidative stress, impairing mitochondrial activity, and diminishing the production of proteins essential for sperm movement (125). A multi-Centre study in China examining 78,952 semen samples from 33,234 donors indicated that both thermal extremes negatively impacted sperm parameters, with heat-related anomalies during the warm season significantly diminishing sperm concentration and motility (130). These findings emphasize the susceptibility of spermatogenesis to temperature variations, illustrating the possible effects of global warming on male fertility (131).

### 5.2 Predictive models and future risks

**Figure 4:**
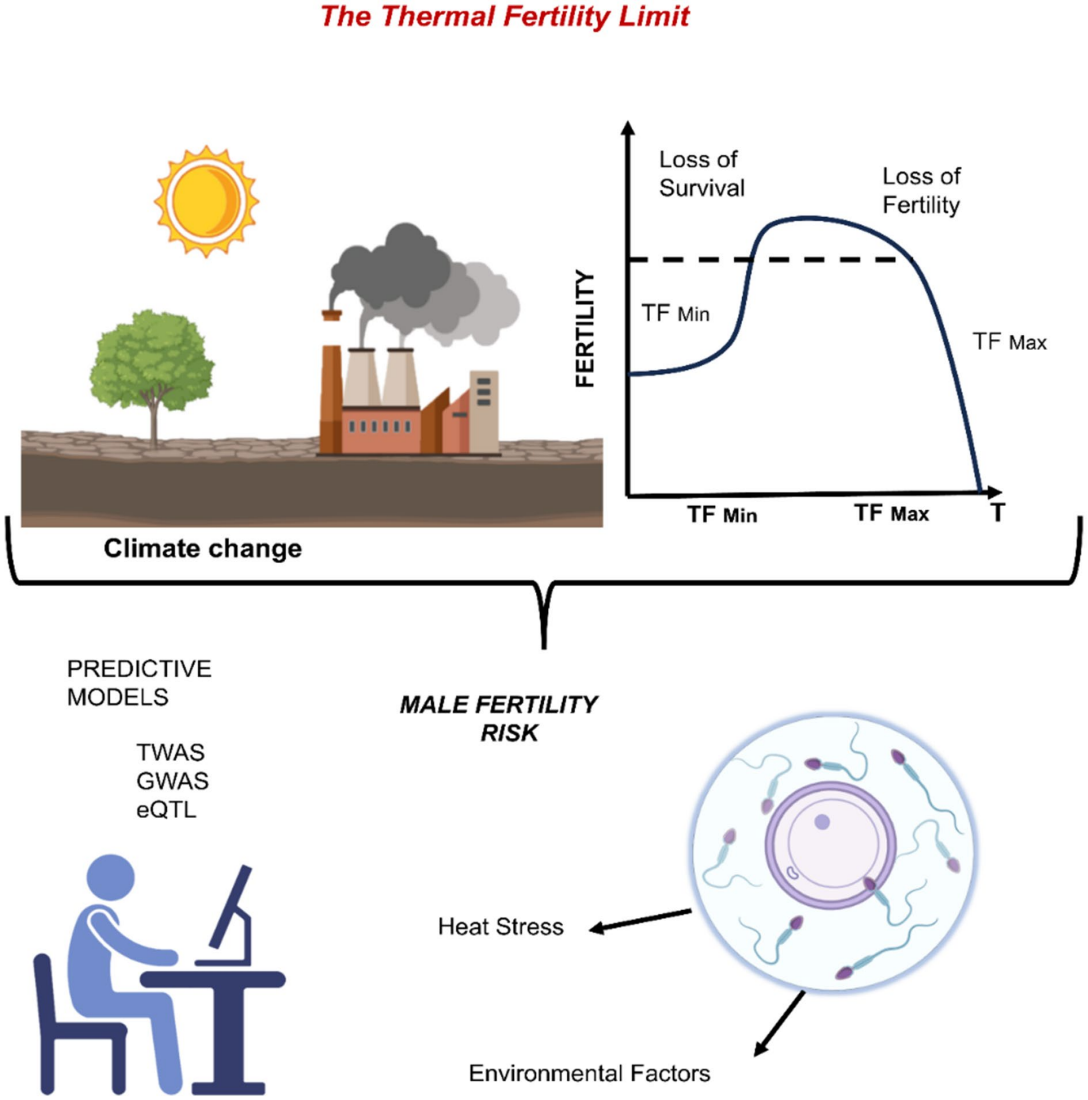
The “thermal fertility limit” concept: integrating climate-driven heat stress with genetic-risk prediction for male fertility.

Effective measures require political will, financial resources, and international cooperation to implement laws on ongoing greenhouse gas emissions, while individuals can initiate or endorse climate-friendly mitigation efforts (132). Advanced prediction models capable of accurately forecasting biological and ecological responses to current and anticipated future climate conditions must be incorporated to evaluate the long-term impacts of climate change on populations (133). The temperature range at which reproductive capacity is forfeited is referred to as the Thermal Fertility Limit (TFL), a broader counterpart to the Critical Thermal Limit (CTL) (134). The CTL delineates the upper and lower temperature limits beyond which an organism cannot sustain physiological activities, typically resulting in mortality. Conversely, TFL concentrates exclusively on fertility loss. While survival remains unchanged, it includes both upper (TF Max) and lower (TF Min) thresholds beyond which fertility is not sustained (135). The TFL functions as a comprehensive framework for unifying the effects of heat stress on reproductive function across several species (Figure 4). The significant distinction between the thermal thresholds for reproduction and survival is underscored by the observation that fertility may be impaired at temperatures far lower than those that induce mortality (136).

High-resolution climate models coupled with multi-omics predictive frameworks transcriptome-wide association studies (TWAS), genome-wide association studies (GWAS) and expression quantitative-trait-loci (eQTL) mapping can forecast individual susceptibility by integrating baseline environmental exposures with host genetics. These algorithms may ultimately identify males at increased risk of heat-induced subfertility and guide personalized mitigation actions. (Abbreviations: TF, thermal fertility; TWAS, transcriptome-wide association study; GWAS, genome-wide association study; eQTL, expression quantitative trait locus).

Identifying this distinction is crucial for accurately assessing a species’ vulnerability to climate change and for informing more targeted conservation and management strategies (137). Predictive models that incorporate transcriptome-wide association studies (TWAS), genome-wide association studies (GWAS), and expression quantitative trait loci (eQTL) mapping are dependable instruments for assessing the effects of climate change on infertility and other reproductive health outcomes (138). These models offer a data-driven basis for evaluating genetic and regulatory elements vulnerable to heat stress in the context of changing environmental conditions. It offers insight into elevated temperatures that may impact male fertility by modifying genes and transcriptomes at critical developmental stages. By identifying specific genes and regulatory networks linked to heat-induced sterility, these models enhance the understanding of biological pathways that are temperature-sensitive (139). Comparable techniques may be employed to identify individuals or communities that have genetic susceptibility to reproductive complications under thermal stress in humans (140). This prediction ability is essential for determining the long-term reproductive risks associated with global warming and for guiding targeted therapies to maintain fertility among populations (141). Predictive genomics is therefore a promising tool for tackling new climate-related risks for human reproductive health. Recent breakthroughs in artificial intelligence (AI) have markedly improved our capacity to forecast male fertility risks through the integration of environmental, behavioral, and biological data (142). A 2024 study presented an explainable AI model that used Extreme Gradient Boosting (XGB) in conjunction with the Synthetic Minority Over-sampling Technique (SMOTE) to forecast male fertility based on adjustable lifestyle and environmental variables (143, 144). This model attained elevated accuracy and offered clear insights into the influence of certain exposures, including heat stress and endocrine-disrupting chemicals, on fertility outcomes (145). Furthermore, studies have indicated that environmental variables such as heavy metals, phthalates, electromagnetic radiation, and temperature can induce sperm DNA damage, resulting in epigenetic alterations that hinder spermatogenesis (7, 146). These findings highlight the imperative for predictive models that evaluate present reproductive status while also forecasting future risks by accounting for both direct environmental influences and heritable epigenetic alterations. Incorporating these models into practice may enable early interventions and guide public health measures to alleviate the negative impacts of environmental variables on male reproductive health (147).

## 6 Future directions in reproductive health

The increasing intricacy of environmental contamination has resulted in the recognition of developing toxins that were once unmonitored or undervalued in their effects on human health (148). Pharmaceuticals and personal care products (PPCPs), toxins resulting from e-waste, and novel endocrine-disrupting substances have been identified as possible hazards to male reproductive health (149). As scientific comprehension advances, the incorporation of sophisticated technologies such as artificial intelligence and machine learning is becoming progressively vital for predicting, evaluating, and mitigating these dangers (150). Notwithstanding progress in Assisted Reproductive Technology (ART), the fundamental reasons of infertility, particularly male-related factors such as age, lifestyle, and epigenetic alterations, are frequently neglected (151). Contemporary predictive models fail to incorporate essential biological and environmental data. A multidisciplinary strategy that integrates biostatistics, machine learning, and sperm epigenome sequencing with clinical and lifestyle data may improve reproductive diagnostics and personalized treatment strategies (152). Studies should investigate if chronic exposure to particulate matter (PM2.5) and cadmium induces a distinct epigenetic signature in sperm DNA, specifically hypermethylation at the MT1A promoter region, which could serve as an early warning biomarker for industrial workers before motility declines. Research must determine whether the reproductive toxicity of microplastics (<5 μm) results from their function as vectors for hydrophobic endocrine disruptors, testing if intratesticular accumulation is associated with mitochondrial ROS production at levels that cannot threshold by physical obstruction (153). The field should move toward AI-driven risk stratification by developing models that integrate local Air Quality Life Index (AQLI) data with baseline sperm DNA fragmentation to predict ART success rates (154). Long-term follow-up studies, with samples taken before and after reducing harmful exposures or targeted therapy, can show if exposure-related molecular changes return to normal.

### 6.1 Integrative biomarkers for reproductive diagnostics

Reproductive health is a vital component of both social and individual well-being, and the development of dependable biomarkers is crucial for detecting various reproductive illnesses (155). Recent research breakthroughs have underscored the significance of integrative biomarkers that amalgamate several biological methodologies to improve diagnostic precision (156). Integrative biomarkers encompass several biological indicators, including as epigenetic, proteomic, genomic, and metabolomic profiles, which might elucidate the fundamental mechanisms of reproductive health and disease (157). Identified a new array of circRNAs that may serve as diagnostic biomarkers for male infertility and offers molecular insights that could enhance diagnostic and treatment strategies (158). Furthermore, research conducted by Soubry (159) indicated that DNA methylation patterns in sperm may function as possible biomarkers for male infertility. This discovery highlights the necessity for a holistic strategy that incorporates both genetic and epigenetic elements in evaluations of reproductive health (159). The amalgamation of several biomarker categories presents a potential opportunity for the advancement of reproductive diagnostics. Integrating genetic, epigenetic, proteomic, and metabolomic data enables healthcare professionals to attain a more thorough comprehension of reproductive health. Ongoing research in this domain is crucial for the advancement of improved diagnostic instruments that can enhance patient outcomes (160, 161).

### 6.2 Artificial intelligence and machine learning approaches

Developments in artificial intelligence (AI) and machine learning (ML) have created fresh opportunities for evaluating and forecasting male fertility hazards linked with environmental exposures (162). One important advancement is the Mojo AISA (Artificial Intelligence Semen Analysis) system, which effectively analyses semen samples using artificial intelligence and deep learning algorithms. By automating sperm parameter evaluation including concentration, motility, shape, and viability, this method lowers human error and inter-observer variability (163). Mojo AISA provides a complete evaluation of sperm quality by including sample preparation, imaging, and analysis into a single automated process, therefore enabling early identification of reproductive problems and guiding therapy plans (164). Additionally, some models utilize deep learning techniques to analyze sperm images and estimate DNA fragmentation index (DFI) values, offering a non-invasive and accurate alternative to traditional assays (165). Such AI-assisted evaluations have demonstrated strong correlations with manual assessments, indicating their potential in clinical settings (166). Emerging studies has increasingly approached the role of AI in the clinical management of male infertility, highlighting its applications in diagnosing hypogonadism, analyzing semen parameters, and predicting outcomes of assisted reproductive technologies (167). It advocates for the integration of AI into clinical workflows to enhance diagnostic accuracy and treatment personalization. A systematic review highlighted the efficacy of AI algorithms in sperm selection, reporting that convolutional neural networks (CNNs) achieved up to 90.73% accuracy in automating morphological classification of sperm images (168, 169). These findings highlight the potential of AI to enhance objectivity and efficiency in sperm analysis. Furthermore, AI algorithms have been applied to predict Johnsen scores, which assess spermatogenic potential based on testicular histology. By analyzing histological images, these models can classify Johnsen scores with high accuracy, aiding in the diagnosis and management of male infertility (170). Machine learning models, including gradient boosted trees and logistic regression, have been employed to predict the presence of spermatozoa in testicular biopsies of patients with non-obstructive azoospermia (NOA). These models achieved Area Under the Receiver Operating Characteristic Curve (AUC-ROC) up to 0.807, aiding in clinical decision-making for sperm retrieval procedures (171). A recent study introduced a deep learning model utilizing VGG-16 architecture to predict semen parameters such as sperm concentration, motility, and morphology based on testicular ultrasonography images. The model achieved area under the receiver operating characteristic curve values of 0.76 for oligospermia, 0.89 for asthenozoospermia, and 0.86 for teratozoospermia, indicating its potential as a non-invasive diagnostic tool (172). Although AI- and ML-based systems and deep-learning semen analysis platforms promise diagnostic accuracy, they have limitations on clinical adoption. Most models are trained on relatively small, single-center datasets, which raise concerns across ethnicities, geographic regions, and laboratory protocols. Deep-learning systems function as “black boxes,” providing limited interpretability, these challenges clinician trust, patient counselling, and regulatory approval. Medicolegal and ethical considerations are unresolved for data privacy, informed consent for AI-assisted decision-making, and algorithmic bias arising from training datasets. The AI output must be updated with current WHO semen analysis guidelines and incorporated into clinician-decision pathways rather than functioning as standalone diagnostics (173). Addressing these challenges is essential for clinical translation of AI/ML technologies in male infertility assessment.

### 6.3 Bridging the evidence gap: strategic human study designs

Animal models and *in vitro* experiments have provided a foundation in understanding microplastics and climate-driven heat stress that compromises reproductive physiology. However, emerging human study shows that follicular fluid contains microplastics, which may pose a risk to reproduction. To verify clinical significance, more human research is required (174). Reducing contact with plastic containers and packaging known to contain endocrine-disrupting chemicals, avoiding high-pollution environments, and exposure to airborne pollutants through indoor air filtration are actionable recommendations based on human evidence in clinical practice. Addressing microplastic toxicity requires detection and precise quantification. This can be done by multi-centric case–control studies on testicular biopsies taken during testicular sperm extraction using Raman or FTIR techniques (175). Studies have linked exposure to synthetic fibers with cancer and respiratory disease in textile workers, but there is still insufficient evidence to directly associate microplastic exposure with specific health problems in humans (176). Animal trials and population-level temperature data have been the primary sources for research on climate-related heat exposure and male fertility. Direct human clinical evidence remains insufficient. Since most existing research depends on wide area temperature averages, one significant challenge is accurately assessing personal heat exposure during the whole spermatogenic cycle (177). Future studies would benefit from personal monitoring devices that quantify heat exposure in daily and occupational environments and relate these measurements to repeated semen analyses. In Japan, temperature changes related to climate change had more male stillbirths, but the reasons are still unclear (178). Certain elements of air pollution can cause health impacts through immunological, inflammatory, and excessive oxidative stress pathways. According to an Australian study, there was a 6.6% increase in emergency room visits during heat waves resulting from PM2.5 exposure (179). Large, long-term cohorts are required to clarify exposure–response relationships and to determine whether heat-related declines in semen quality are transient or persistent.

### 6.4 Strategies for mitigation and intervention

When a significant amount of research focuses on diagnosis, biomarker recognition and predictive modelling, dietary practices should be revised to reduce the Western-style diet, which is highly processed foods that lacks in vitamins. Conversely, eating a diet low in saturated and trans fats and high in antioxidants and anti-inflammatory agents like vitamin C, vitamin E, vitamin D, folate, *β*-carotene, selenium, zinc, cryptoxanthin, and lycopene could prevent male infertility (180). Lifestyle-based approaches are equally important and involve avoiding scrotal hyperthermia, reducing excess body weight, quitting smoking, limiting alcohol consumption, improving sleep quality, and engaging in regular physical activity, all of which help maintain hormonal balance and lower systemic inflammation. According to a recent study, endogenous coenzyme Q10 levels and platelet mitochondrial activity are indicators of mitochondrial health in infertile males (181). Data obtained using such markers may serve as a basis for patient-tailored antioxidant preventive therapy and the long-term evaluation of its effects. For male fertility, the effects of exposure to various environmental and lifestyle factors may be reversible. Using personal protective equipment at workplace, reducing the intake of highly processed and hot foods packed in plastic, and indoor air quality through filtration are examples of practical solutions. Many symptomatic men who stop such exposure exhibit spontaneous improvements in fertility. For example, a recent study found that quitting smoking improved sperm concentration, semen volume, and overall sperm count (182). Additionally, medical treatments such as aromatase inhibitors, gonadotropins, and selective estrogen receptor modulators may be taken into consideration (183). Assisted reproduction is required if neither lifestyle changes nor the non-invasive therapies are successful in increasing male fertility. The current understanding on health risks posed by environmental factors should motivate policymakers to implement legal frameworks that mitigate their adverse effects on human health (184). Moreover, this raises the need for enhanced education of children and adolescents aimed at reducing environmental exposure. In order to lower population-level reproductive risk, policy measures including tighter regulation of endocrine-disrupting chemicals, microplastic management, better air quality standards, and monitoring heavy metal pollution in water and food systems are important. Rather than analyzing semen parameters, therapeutic approaches should be evaluated in well-designed randomized controlled trials (185). AI can assist with diagnosis, individualized care, treatment prediction, and recovery tracking.

## 7 Conclusion

All things considered, this study emphasizes the serious problem of male infertility in contemporary society, which has greatly escalated recently. Affecting almost half of couples worldwide, male infertility is a major proportion of all occurrences of infertility and is usually connected to low semen quality. The multifaceted nature of male infertility is highlighted through its association with various environmental factors like air pollutants, endocrine disrupting chemicals, heavy metals, microplastics, climatic changes. The increase in the environmental quality deterioration because of air pollution, use of pesticides, plastics and other synthetic compounds can be a great hazard to male fertility potential. These environmental factors have been found to be important contributors to the decline in male reproductive health due to their harmful effect on various stages of spermatogenesis and overall sperm function. Increasing awareness, facilitating comprehensive evaluations, and supporting dedicated research initiatives are essential steps toward a deeper understanding and more effective management of male infertility. Although this study provides valuable insights into the environmental contributors to male infertility, it does not specifically address treatment approaches. To improve male infertility care, it is recommended to practice an integrate test for heavy metals, phthalates, bisphenol A (BPA), and microplastics into standard infertility evaluations, particularly for unexplained low or absent sperm counts. To optimize assisted reproductive technology (ART) outcomes, protocols should incorporate individual assessments of environmental exposures such as air pollution, endocrine-disrupting chemicals, heavy metals, and occupational hazards while also considering regional environmental risk factors that may influence sperm quality and fertilization potential. This integration would function as a unified diagnostic workflow where AI algorithms process patient-specific environmental data (eco-profiles) alongside multi-omics, such as sperm DNA methylation patterns and seminal plasma proteomics. Clinicians with these datasets could identify specific toxicological pathways affecting an individual patient, enabling to generate personalized risk scores. This approach would guide targeted interventions such as specific antioxidant therapies or lifestyle modifications prior to assisted ART. In order to understand environmental factors that affect male reproductive health, extensive long-term research is necessary. Future research should explore the specific effects of various environmental pollutants on spermatogenesis, sperm function, and overall fertility. A deeper understanding of these can help to create more personalized and effective treatments, it will be specific to an individual’s environmental exposure index. Establishing therapeutic guidelines that include environmental exposure assessments into standard infertility tests is also essential. Furthermore, while taking careful consideration of the associated ethical and legal implications into account, the incorporation of cutting-edge technologies like AI-assisted diagnostics and customized ART plans based on environmental risk profiles should be investigated.

## Glossary

ART: Assisted Reproductive Technology
WHO: world Health Organization
IVF: Invitro Fertilization
ICSI: Intracytoplasmic sperm injection
EDC: Endocrine disrupting chemicals
HPG: Hypothalamic Pituitary Gonadal
PM: Particulate Matter
PAH: Polycyclic aromatic hydrocarbon
ROS: Reactive oxygen species
TNF: Tumor Necrosis factor
IL: Interleukin
NF: Necrosis factor
LH: Luteinizing hormone
FSH: Follicle-Stimulating hormone
AQLI: Air quality life index
O_₃_: ozone
CO: Carbon monoxide
NO_₂_: Nitrogen dioxide
Cd: Cadmium
Pb: Lead
Cr: Chromium
Hg: Mercury
PVC: Polyvinyl chloride
AAS: Atomic absorption spectroscopy
F-AAS: Flame Atomic absorption spectroscopy
ASPR: Age standardized Prevalence rate
ICP: MS -Inductively coupled mass spectroscopy
MP: Microplastics
TFL: Thermal Fertility Limit
CTL: Critical temperature limit
TWAS: transcriptome-wide association study
GWAS: genome-wide association study
eQTL: expression quantitative trait locus.
